# Vibrational circular dichroism unveils hidden clues

**DOI:** 10.1038/s41377-023-01239-7

**Published:** 2023-08-31

**Authors:** Dang Du Nguyen, Inki Kim

**Affiliations:** 1 Department of Biophysics, Institute of Quantum Biophysics, Sungkyunkwan University, Suwon, 16419 Republic of Korea; 2 Department of Intelligent Precision Healthcare Convergence, Sungkyunkwan University, Suwon, 16419 Republic of Korea

**Keywords:** Metamaterials, Infrared spectroscopy, Imaging and sensing

## Abstract

Infrared chiral plasmonic metamaterials based on perpendicularly positioned nanorods enable surface-enhanced vibrational circular dichroism for more selective and sensitive identification of protein fingerprints and enantioselective sensing, which creates a new pathway for chemical or biomedical applications.

Chirality is a widespread property in life and is present in various biological systems, ranging from fundamental molecules such as amino acids and glucose^1^ to the intricate chiral structures of DNA strands^2^ and proteins^3^. Enantiomers, a common example of chirality, are chiral isomers with opposite handedness that display identical physical and chemical properties because of their similar functional groups and compositions. However, they often manifest distinct levels of toxicity in cellular systems^4^. Therefore, the precise detection of enantiomers is important in analytical chemistry, biomedicine, pharmacology, and toxicology.

Chiroptical spectroscopic solutions have gained prominence in chirality sensing. This is attributed to the interaction between chiral molecules and the spin momentum of circularly polarized light, resulting in an immediate response and accurate detection. Optical tools, such as circular dichroism (CD), are extensively used for the molecular characterization of chiral compounds across ultraviolet, visible, and near-infrared spectral regions. Their applications are particularly focused on discerning the handedness or chirality of molecules. Nevertheless, the molecular CD signals observed at these wavelengths do not provide comprehensive information regarding the molecular chemical structures, primarily because of their limited selectivity. Consequently, the utility of CD-based methods is hampered when it comes to effectively sensing and differentiating various types of chiral molecules present in mixtures. Therefore, there has been a growing focus on using vibrational circular dichroism (VCD) in the mid-infrared (MIR) wavelength range to extract valuable information regarding the chiral properties of molecules. This technique analyzes the chemical bonds and functional groups that act as infrared fingerprints to reveal the molecular structure, particularly in the amide vibrational band for protein anlysis^5^. The primary drawback of VCD spectroscopy is its inherently weak signals, which are three orders of magnitude smaller than those in the visible and ultraviolet ranges.

The MIR nanophotonic platform is a highly promising solution for overcoming the sensing restraints of VCD signals. This effectively addresses this issue by harnessing confined optical fields and resonant coupling mechanisms. Recently, the concept of chirality has been extensively employed in the domain of metamaterials, leading to diverse applications, including chiral bioimaging^6^, especially in sensing^7,8^. Chiral metamaterials comprise subwavelength structures with distinct orientations and handedness. Owing to their anisotropic properties, these structures can interact differently with circularly polarized light. Furthermore, due to the fascinating coupling phenomenon observed between enantiomers and chiral metamaterials, the localized near-field region experiences intense optical chirality^9^. This enhancement leads to a more robust chiral light–matter interaction, thereby sparking increased research attention in sensing applications. Nevertheless, there has been limited focus on enhancing weak VCD signals^10^, and existing studies have not proposed comprehensive design and optimization methods for MIR chiral metamaterials. Consequently, there is a lack of robust chiral light–matter interactions within the near field. Developing advanced VCD sensors that can accurately calibrate signals with smaller sample volumes is crucial for unlocking the potential of VCD for selectively detecting chiral mixtures with different concentrations and enantiomeric ratios.

Now, writing in this issue of *Light: Science & Applications*, Cheng Xu and colleagues at the National University of Singapore and the Institute of Microelectronics (IME), Agency for Science, Technology, and Research (A*STAR), Singapore, have demonstrated surface-enhanced vibrational circular dichroism (SEVCD) spectroscopy for the improvement of molecular IR and CD signal sensing using infrared chiral plasmonic metamaterials (IRCPM) consisting of gold (Au) nanorods on top of an Al_2_O_3_-Au-Si structure (Fig. 1) to satisfy the aforementioned requirements^11^. In this study, to investigate the mechanism of the enhanced VCD sensing signal, reflective chiral metamaterials with two mutually orthogonal resonant modes were used. Following a meticulous analysis of the molecular interaction and the near-field coupling among these resonant structures, they proceeded to fine-tune their structure to extract a significantly enhanced far-field molecular signal and harness this platform as a biosensor to detect protein secondary structures.

**Fig. 1:**
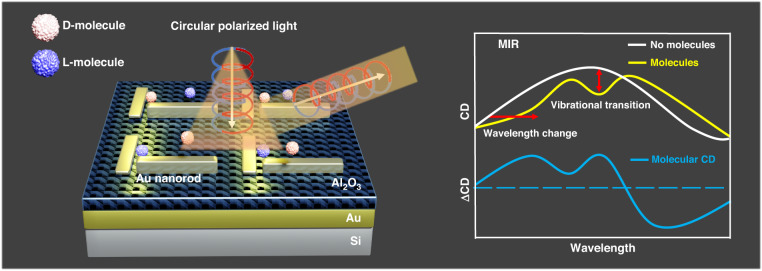
Schematic of the enhancement of a VCD signal via the IRCPM platform. In addition to wavelength change, the MIR regime also facilitates the occurrence of molecular vibrational transitions, and the corresponding VCD signal can supply valuable information regarding vibration and chirality

This study proposes a design framework that incorporates a loss engineering method, underpinned by temporal coupled-mode theory, to facilitate the design and optimization of chiral metamaterials. This approach involves a thorough investigation of the loss ratio and near-field coupling coefficients, which influence the absorption disparity between left- and right-handed circularly polarized electromagnetic waves. Through deliberate manipulation of these parameters, chiral metamaterials were optimized by adjusting both the in-plane and out-of-plane asymmetry factors. This strategic tuning aimed to enhance the VCD signal, resulting in a larger and more pronounced response.

The developed SEVCD chiral molecule-sensing platform demonstrated excellent performance by achieving a substantial six-magnitude signal enhancement and exhibiting superior selectivity for chiral molecules compared to traditional VCD spectroscopy. Moreover, this platform showcased an impressive limit of detection reaching ~23 zeptomoles in protein-sensing applications for a small sample volume of 1 μL. Additionally, it enabled the enhanced VCD sensing of mixed protein secondary structures, offering exceptional selectivity through vibrational transitions.

The ability to discern distinct chiral structures within mixtures can enhance the application ranges for chiral sensors, particularly in sectors such as the pharmaceutical industry. This is relevant in situations where chiral impurities present during drug production can induce adverse side effects or produce contrasting effects^12^. Furthermore, the SEVCD chiral-sensing platform improved molecule-sensing signal and broadened the range of extractable information, enabling label-free and compact on-chip molecular identification for clinical diagnosis^13^ across diverse species and small sample volumes^14^.
